# Morphological evolution, growth mechanism, and magneto-transport properties of silver telluride one-dimensional nanostructures

**DOI:** 10.1186/1556-276X-8-356

**Published:** 2013-08-20

**Authors:** GaoMin Li, XiaoBing Tang, ShaoMin Zhou, Ning Li, XianYou Yuan

**Affiliations:** 1 Key Laboratory for Special Functional Materials of Ministry of Education, Henan University, Kaifeng 475004, People's Republic of China; 2 Department of Biology and Chemistry, Hunan University of Science and Engineering, Yongzhou, Hunan 425100, People's Republic of China

**Keywords:** Silver telluride, One-dimensional nanostructures, Morphological evolution, Growth mechanism, Magneto-transport properties

## Abstract

Single crystalline one-dimensional (1D) nanostructures of silver telluride (Ag_2_Te) with well-controlled shapes and sizes were synthesized via the hydrothermal reduction of sodium tellurite (Na_2_TeO_3_) in a mixed solution. The morphological evolution of various 1D nanostructures was mainly determined by properly controlling the nucleation and growth process of Ag_2_Te in different reaction times. Based on the transmission electron microscopy and scanning electron microscopy studies, the formation mechanism for these 1D nanostructures was rationally interpreted. In addition, the current–voltage (*I*-*V*) characteristics as a function of magnetic field of the highly single crystal Ag_2_Te nanowires were systematically measured. From the investigation of *I*-*V* characteristics, we have observed a rapid change of the current in low magnetic field, which can be used as the magnetic field sensor. The magneto-resistance behavior of the Ag_2_Te nanowires with monoclinic structure was also investigated. Comparing to the bulk and thin film materials, we found that there is generally a larger change in *R* (*T*) as the sample size is reduced, which indicates that the size of the sample has a certain impact on magneto-transport properties. Simultaneously, some possible reasons resulting in the observed large positive magneto-resistance behavior are discussed.

## Background

During the past few decades, a shape-controlled synthesis of semiconducting crystals with well-defined morphologies, such as belts, wires, rods, tubes, spheres, sheets, combs, and cubes, has attracted considerable attention due to their novel properties and applications in many fields [1-7]. Among these nanostructures, one-dimensional (1D) nanostructures have increasingly become the subject of intensive research due to their potential applications in a variety of novel devices [8-10]. The most prominent example is certainly the carbon nanotubes [11,12]. Not only that, considerable efforts have been spent on the synthesis of nanobelts, nanowires (NWs), and other 1D nanostructures. Especially, with the miniaturization of devices in the future, searching for interconnects remains a challenge to future nanoelectronics. Therefore, it is essential to investigate 1D nanomaterials which can be applied in the nanoscale field.

As one typical example of the silver chalcogenides, Ag_2_Te has attracted increasing attention due to its much more technological prospects [10,13,14]. As reported, Ag_2_Te can transfer its structural phase from the low-temperature monoclinic structure (β-Ag_2_Te) to the high-temperature face-centered cubic structure (α-Ag_2_Te) at about 145°C [15,16]. Low-temperature β-Ag_2_Te is a narrow band gap semiconductor with high electron mobility and low lattice thermal conductivity [17], which is desirable for its high figure of merit for thermoelectric applications. In the α-Ag_2_Te phase, silver cations can move freely, which enhance the conductivity, leading to superionic conductivity [15]. More recently, it has been reported that Ag_2_Te is a new topological insulator with an anisotropic single Dirac cone due to a distorted antifluorite structure [14], leading to new applications in nanoelectronics and spintronics. It is also known that a huge large positive magneto-resistance (MR) has been observed in the case of silver telluride bulk samples [18] or thin films [19]. However, to the best of our knowledge, the MR behavior of Ag_2_Te nanostructured materials is rarely reported. Here, we systematically investigate the current–voltage (*I*-*V*) characteristics under different magnetic fields and the extraordinary MR behavior of Ag_2_Te nanowires. The magneto-resistance can be strongly affected by the details of the Fermi surface geometry and character of electron–electron (e-e) interactions [20] and therefore gives valuable insight into the physics dominating the conductivity. Furthermore, Ag_2_Te with nontrivial MR can provide great opportunities in magnetic sensor and memory applications.

It was reported that Ag_2_Te tended to form 1D nanostructures. For instance, the rod-like structure of Ag_2_Te was synthesized by the method based on the template-engaged synthesis in which the Te nanorods were used as template reagents [21]. Ag_2_Te nanotubes have been synthesized hydrothermally when sodium tellurite (Na_2_TeO_3_) and silver nitrate (AgNO_3_) in hydrazine/ammonia mixture were autoclaved at 393 K [22]. Ag_2_Te NWs were obtained by cathodic electrolysis in dimethyl sulfoxide solutions containing AgNO_3_ and TeCl_4_ using porous anodic alumina membrane as the template [17]. Recently, Ag_2_Te NWs were synthesized by a composite hydroxide-mediated method, where AgNO_3_ and Te powder were heated at 498 K in a Teflon vessel containing ethylenediamine and hydrazine hydrate [23]. Samal and Pradeep [24] have developed a room-temperature solution-phase route for the preparation of 1D Ag_2_Te NWs. In addition, our research group has more recently reported the synthesis and electrical properties of individual Ag_2_Te NWs via a hydrothermal process [25]. Herein, on this basis, we demonstrate a simple hydrothermal method for the synthesis of Ag_2_Te 1D nanostructures by employing ammonia acting as a complexing reagent and pH regulator hydrazine hydrate (N_2_H_4_ · H_2_O) acting as a reducing reagent. Very interestingly, we discovered the morphological evolution during the formation of 1D NWs. The morphological evolution for the 1D nanostructures is considered as the desired agent for understanding the growth mechanism and formation kinetics of crystals [26-28]. Therefore, we believe that this discoveryof the formation of 1D Ag_2_Te nanostructures could promote further studies and potential applications.

## Methods

The materials used include Na_2_TeO_3_, AgNO_3_, aqueous hydrazine solution (80%) (N_2_H_4_ · H_2_O), and ammonia (25%) (NH_3_ · H_2_O). All of the reagents used in the experiment were directly used without further purification. The preparation of Ag_2_Te nanostructures involved a hydrothermal process as our previous works [25]. In a typical experiment, 0.5 mmol of Na_2_TeO_3_ and 1.0 mmol of AgNO_3_ were dissolved in 15 mL of deionized water. After stirring for minutes, 0.40 mL of N_2_H_4_ · H_2_O (80%) and 0.40 mL of NH_3_ · H_2_O (25%) were dropped in the solution. A mixed solution was obtained and then transferred into a 25-mL Teflon-lined stainless steel autoclave, followed by heating at 160°C for a period of time in an electric oven. After heating, the autoclave was cooled down naturally to room temperature. After the hydrothermal treatment, the precipitate was collected and rinsed with distilled water and ethanol and then dried in air for further characterization. After a serious treatment, the as-synthesized sample was obtained for further characterization.

The size and morphology of the as-synthesized Ag_2_Te nanostructures were characterized using scanning electron microscopy (SEM) (JEOL JSM5600LV, Akishima-shi, Japan), equipped with X-ray energy dispersive analysis spectrum (EDS). The crystalline structure and chemical composition were characterized by transmission electron microscopy (TEM) and high-resolution TEM (HRTEM) and selected area electron diffraction (SAED) (JEOL 2010, operated at an accelerating voltage of 200 kV). X-ray photoelectric spectrum (XPS) (Kratos AXIS Ultra, Kratos Analytical, Ltd., Manchester, UK) and X-ray diffraction (XRD) (X’pert MRD-Philips, Holland). Thermogravimetric and scalable differential thermal analysis (TG-SDTA) was carried out at a heating rate of 10°C min^−1^ in N_2_ gas at a flowing rate of 50 mL min^−1^ using a TGA/SDTA851e system. The room-temperature Raman spectra of the Ag_2_Te NWs were recorded with a micro-Raman spectrometer (Renishaw 1000, Wotton-under-Edge, UK) equipped with a CCD detector and an Ar^+^ laser with a 514.5-nm excitation line (diameter of laser spot, 3 μm) and 4.2 mW of power. The MR of these device measurements were carried out at room temperature using a Quantum Design 9 T physical property measurement system (PPMS) with a rotational sample holder.

## Results and discussion

**Figure 1:**
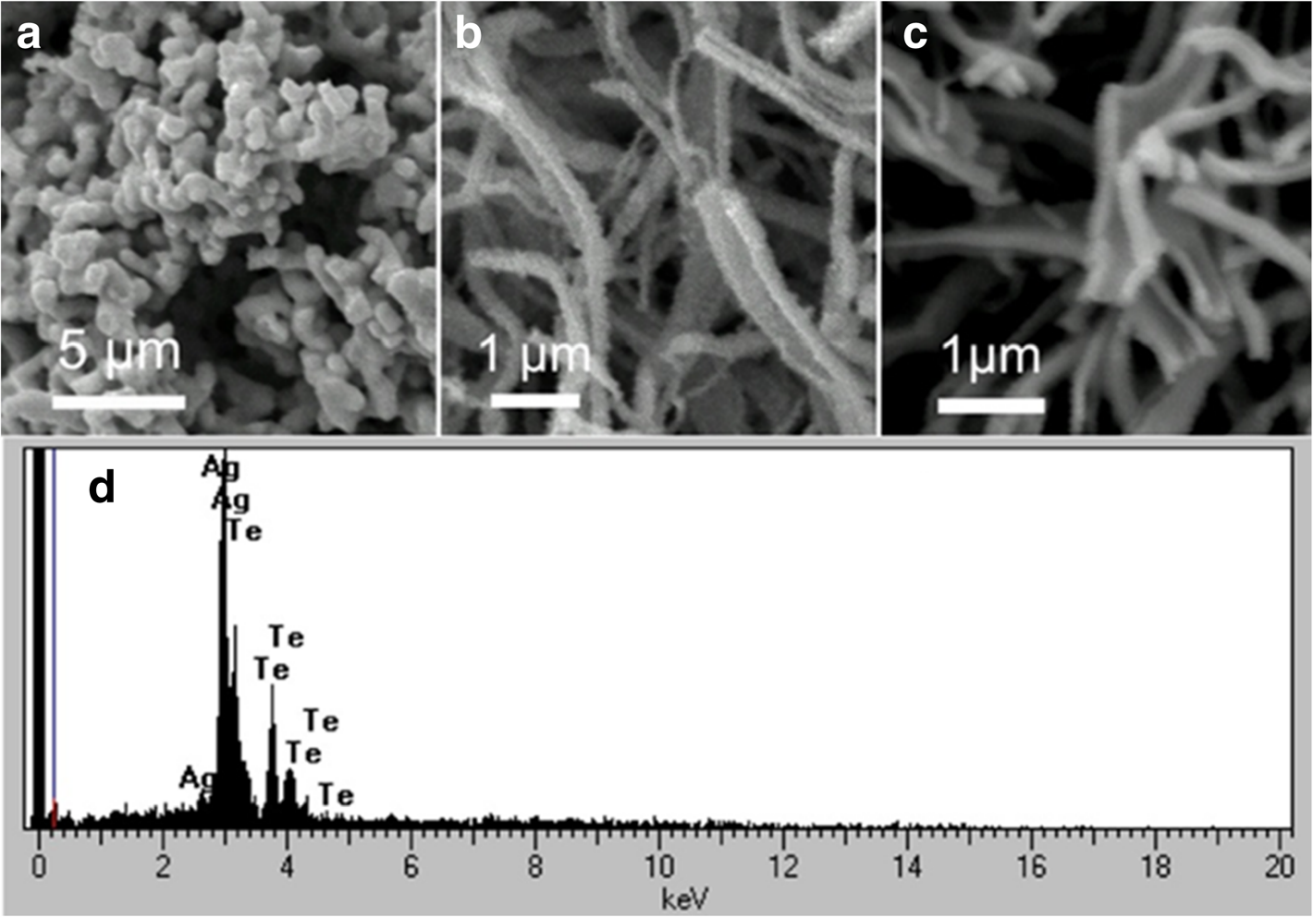
**Morphology evolution sequence of Ag**_**2**_**Te products as different reaction durations.** The SEM images of the as-prepared Ag_2_Te products under different reaction times at 160°C: **(a)** 0, **(b)** 3, and **(c)** 6 h. **(d)** EDS of the Ag_2_Te nanobelts.

The morphology evolution of hydrothermal treatment of Ag_2_Te samples under different reaction times at 160°C is displayed in Figure 1. From Figure 1a, we clearly see that the Ag_2_Te sample exists in the form of a particle before heating. After 3 h of reaction time, some narrow and thin nanobelt structures (Figure 1b) begin to appear. When heated for 6 h, the sample further curls and grows into nanobelt regularly as obviously observed in Figure 1c. In addition, The EDS of the as-synthesized Ag_2_Te nanobelts is shown in Figure 1d. According to the quantification of the EDS peaks, the atomic ratio of Ag to Te is 43:22, close to the stoichiometry of Ag_2_Te, which confirmed a stoichiometric composition of the Ag_2_Te products. The XRD spectra of the Ag_2_Te products under various growth times (3, 6, and 12 h reaction time) are shown in Additional file 1: Figure A1.

**Figure 2:**
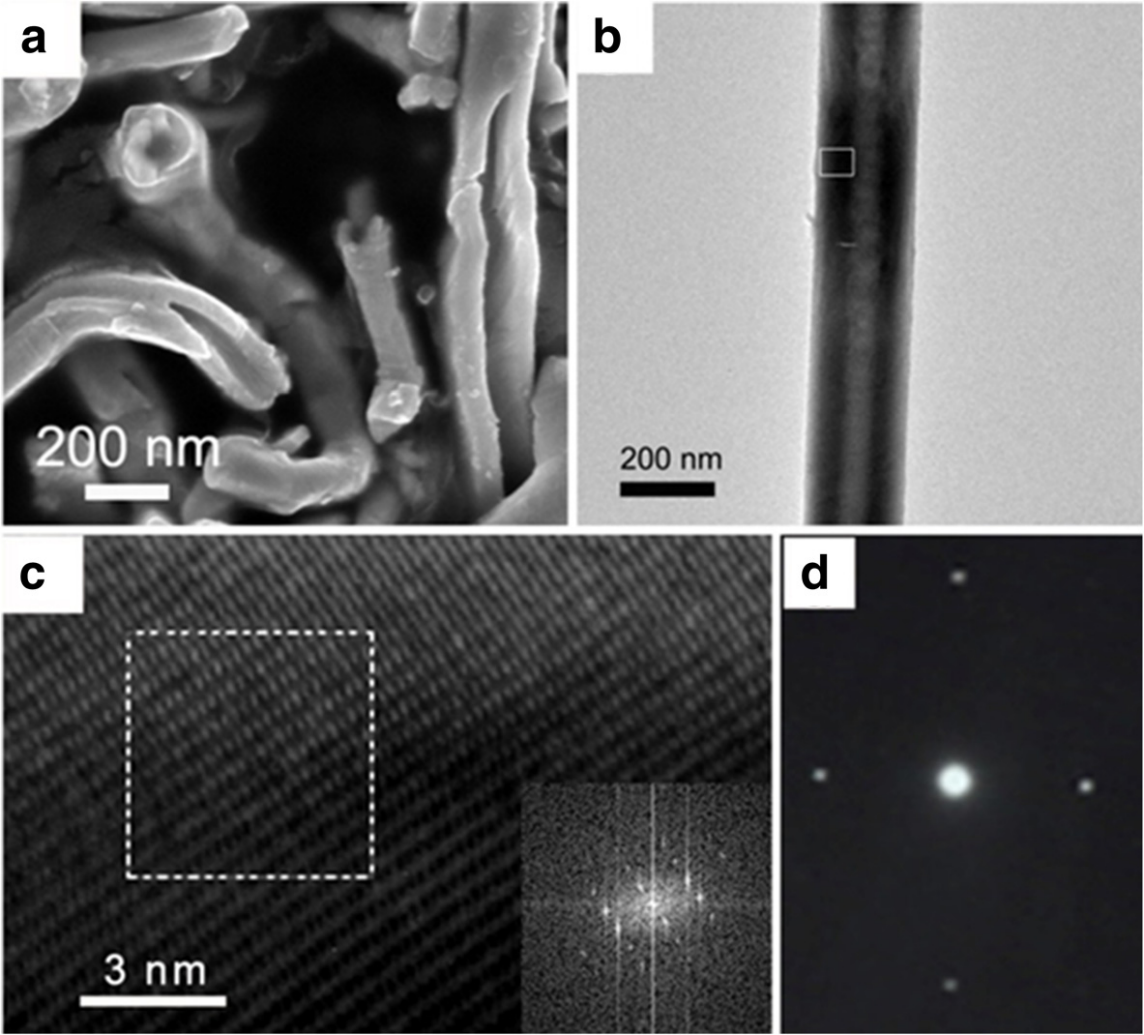
**The morphology and structure of the Ag**_**2 **_**Te nanotubes. (a)** The high magnification SEM image of the as-prepared Ag_2_Te nanotubes. **(b)** TEM image of the single Ag_2_Te nanotube. **(c)** HRTEM image recorded from the black square in **(b)** and FFT image (inset). **(d)** SAED patterns of the single Ag_2_Te nanotube.

The morphology and structure of the Ag_2_Te nanotubes were examined with SEM and TEM. The SEM image (Figure 2a) of the Ag_2_Te nanotubes shows that the product obviously presents tubular structures which have been rolled into tubes or half-pipes. As can be seen from the image, the nanotubes have lengths of several microns and outer diameters of 100 to 230 nm. Figure 2b is a TEM image of a single Ag_2_Te nanotube. The TEM image further provides that the product is tubular with an approximately 80 nm of tube wall in thickness. In addition, we can obviously see that the outer diameter of the tube is approximately 200 nm. The high-quality crystal structure of Ag_2_Te nanotubes is demonstrated in a HRTEM image shown in Figure 2c, where abruptness at an atomic level can be confirmed and no defects are observed. The lattice spacing between the atomic planes was determined to be 0.56 nm in accordance with the distance between layers, indexed to the monoclinic Ag_2_Te phase. Correspondingly, the fast Fourier transform (FFT) pattern (inset in Figure 2c) shows obvious single crystalline nature and can be easily indexed to the cubic structure. The corresponding SAED pattern in Figure 2d can be indexed to the crystal of Ag_2_Te, which further provides strong evidence for confirming single crystalline growth in the fine monoclinic crystal structure.

**Figure 3:**
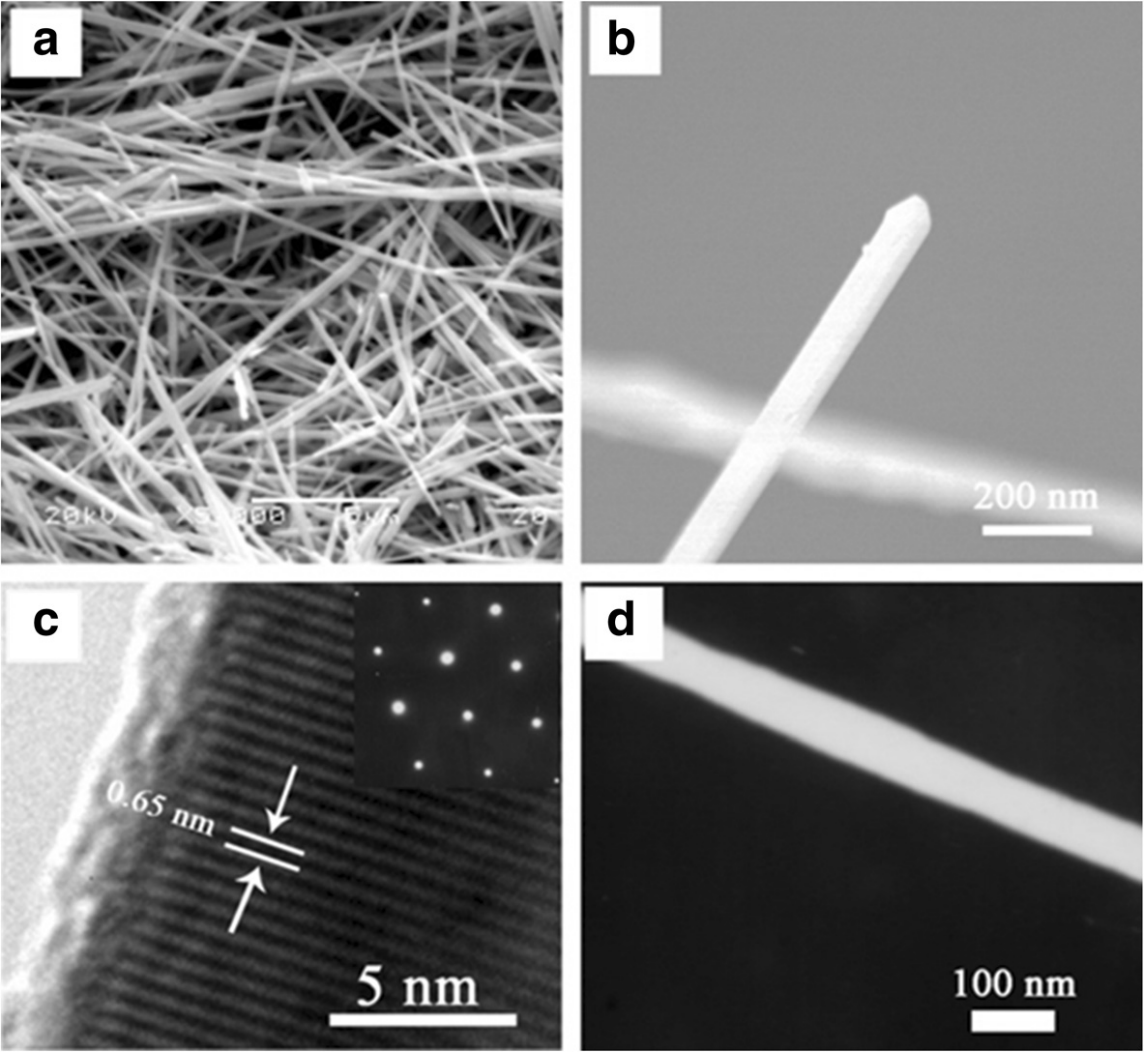
**The morphology and structure of the Ag**_**2 **_**Te nanowires. (a)** The SEM image of the as-prepared Ag_2_Te nanowires synthesized at 160°C for 24 h. **(b)** HRSEM image of a single Ag_2_Te nanowire. **(c)** HRTEM image of a single Ag_2_Te nanowire, and the upper right inset for the corresponding SAED pattern. **(d)** TEM of a single Ag_2_Te nanowire.

The morphology and structure of the Ag_2_Te nanowires were examined with SEM in Figure 3a. Numerous long straight nanowires are formed, and all of the nanowires are demonstrated with the relatively uniform diameter about 200 nm and a typical length of tens of micrometers. A detailed investigation was performed using high-magnification SEM (HRSEM)/HRTEM/TEM. Figure 3b shows a typical high-magnification SEM image of the single Ag_2_Te nanowire with diameters about 150 nm and lengths ranging from 8 to 10 μm. A typical HRTEM image (Figure 3c) taken from a small square in Figure 3b demonstrates clear lattice fringes with an interplanar spacing of 0.65 nm. Moreover, a representative SAED (upper right inset in Figure 3c, taken from a small square in Figure 3b, too) further substantiates that the Ag_2_Te nanowire has a single crystalline structure with a monoclinic phase. Further, according to the quantification of XPS peaks shown in Additional file 2: Figure A2, the molar ratio of Ag to Te is 2.08:1.00, which is close to the stoichiometry of Ag_2_Te. To further ascertain the chemical compositions of the nanowires, the as-prepared products were examined by TG-SDTA and Raman scattering spectroscopy in Additional file 3: Figure A3 and Additional file 4: Figure A4, respectively.

**Figure 4:**
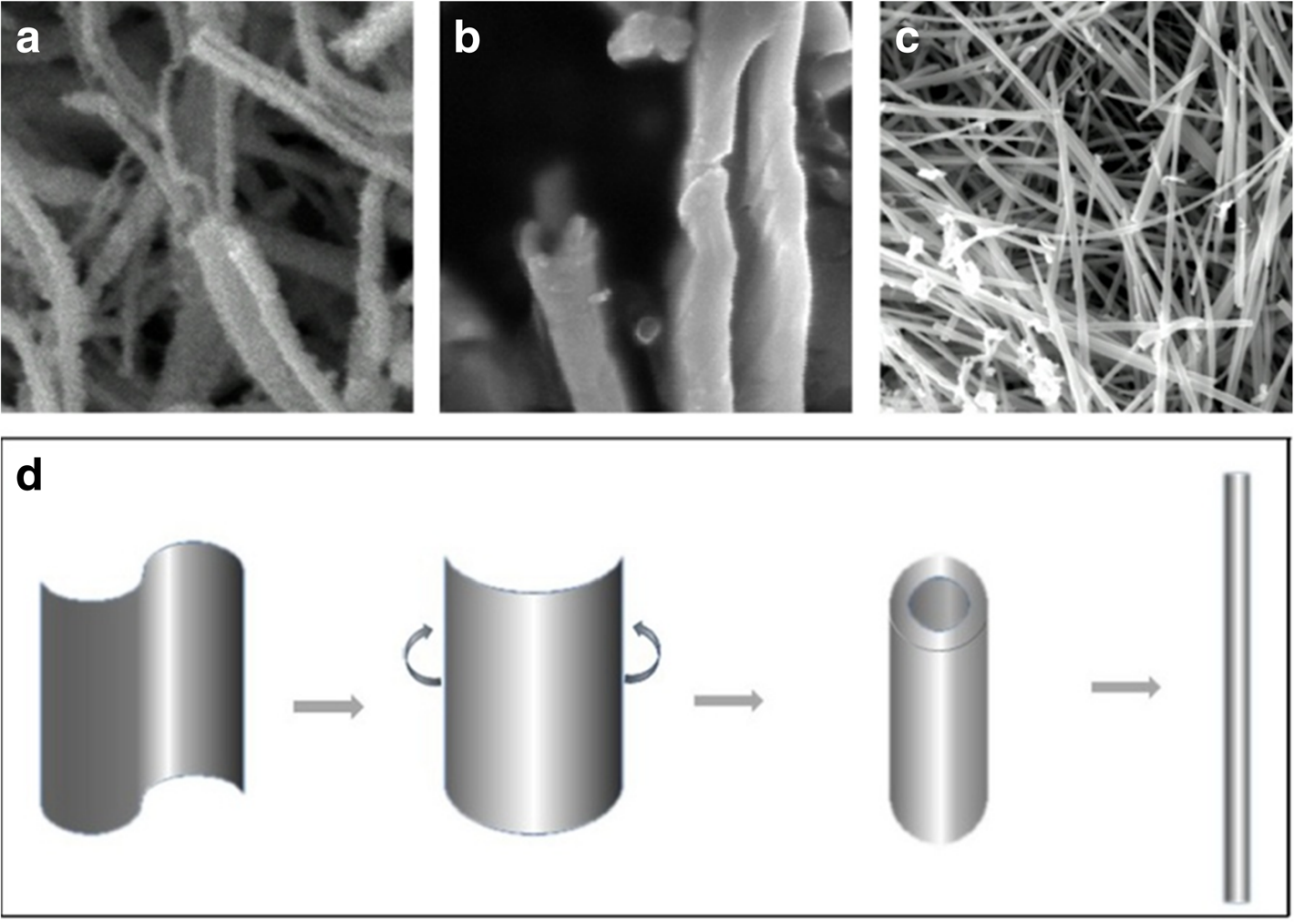
**The morphology evolution sequence and schematic diagrams of the formation of Ag**_**2 **_**Te nanowires and nanostructures. (a, b, c)** Morphology evolution sequence of the formation of Ag_2_Te nanowires. **(d)** The schematic diagrams of the formation of Ag_2_Te nanostructures: nanobelt, nanotube, and nanowire.

To further obtain a complete view of the Ag_2_Te ultra-long and straight NW formation process and its growth mechanism, the detailed time-dependent evolution of the morphology was evaluated by SEM (Figure 4a,b,c). As shown in Figure 4a, when the hydrothermal reaction proceeded for 3 h, the products are mainly composed of Ag_2_Te nanobelts or half-nanotubes. If the reaction time is increased to 12 h, these Ag_2_Te nanobelts further curled up along the axis, became half-tubes, and finally grew into nanotubes (Figure 4b). When the reaction time was increased to 24 h, the Ag_2_Te nanotubes grew into NWs with a diameter of about 100 to 200 nm and a typical length of tens of micrometers eventually. Based on the above experimental observations, a plausible formation mechanism of the Ag_2_Te ultra-long NWs is proposed (Figure 4d). We believe that the formation process of the ultra-straight and long Ag_2_Te NWs could be rationally expressed into three sequential steps: (1) the formation of Ag_2_Te nanobelts and the existence of half-tube structures at an early stage, (2) the nanobelts further curled up along the axis, became half-tubes, and finally grew into nanotubes via the rolling-up mechanism [22,28], (3) with the extended reaction time, Ag_2_Te nanotubes continue to grow and grow into NWs eventually. On the basis of the experimental results and discussion, and according to previous reports [22,25], a possible mechanism for the formation of ultra-straight and long Ag_2_Te NWs may be explained by the following reactions:

(1) $${\mathrm{TeO}}_{3}^{2‒}{+N}_{2}H_{4}\to {\mathrm{Te}+N}_{2}{+H}_{2}O$$

(2) $${\mathrm{Te}+N}_{2}H_{4}{+\mathrm{OH}}^{‒1}\to {\mathrm{Te}}^{2‒}{+N}_{2}{+H}_{2}O$$

(3) $${2\mathrm{Ag}}^{+}{+\mathrm{Te}}^{2‒}{=\mathrm{Ag}}_{2}\mathrm{Te}$$

**Figure 5:**
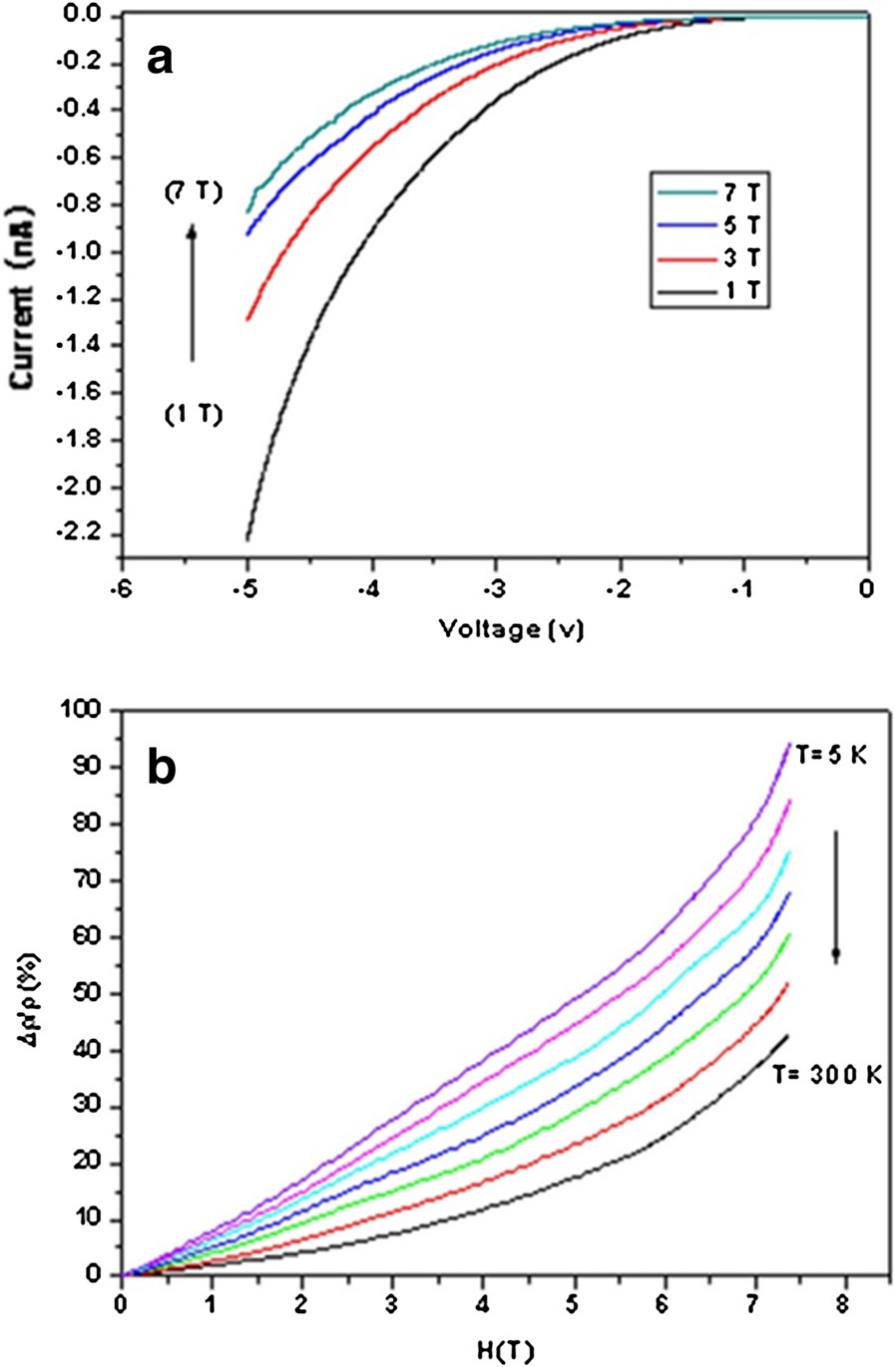
***I-V *characteristics of the Ag**_**2 **_**Te nanowires at room temperature and normalized magneto-resistance for Ag**_**2 **_**Te nanowires. (a) ***I*-*V* characteristics of the Ag_2_Te nanowires at room temperature under a series of magnetic field, *B* = 1, 3, 5, and 7 T; **(b)** the normalized magneto-resistance Δ*ρ* (*T*, *H*) / *ρ* (*T*, *H*) for Ag_2_Te nanowires as a function of magnetic field *H* at a series of temperatures *T* = 5, 10, 20, 40, 80, 160, and 300 K.

To investigate the magneto-transport properties of Ag_2_Te NWs, PPMS measurements were carried out. *I*-*V* characteristics of the nanowires at room temperature as a function of magnetic field (*B* = 1, 3, 5, and 7 T) are shown in Figure 5a. The black curve is the *I*-*V* of the magnetic field of 1 T. Obviously, the current increases nonlinearly with the increasing voltage. Without changing the other experimental conditions, only changing *B* to 3 T, the *I*-*V* of the Ag_2_Te sample (red line) displays a smaller absolute value of the corresponding current and a larger resistance at the same voltage conditions. When the magnetic field is adjusted to 5 and 7 T (the blue and the green line), respectively, the absolute value of the current continues to decrease at the same voltage conditions. It is noteworthy that from Figure 5a, we can clearly see that Δ*I* from 1 to 3 T is larger than that from 3 to 7 T where the voltage is −4 V. That is to say, the *I*-*V* of Ag_2_Te sample is more sensitive at low magnetic field. This phenomenon reveals that the Ag_2_Te nanowires are suitable for low magnetic field sensor. In addition, the magneto-resistance curves under different temperature conditions are illustrated in Figure 5b. The MR was calculated as MR = (*ρ*_*H*_ − *ρ*_0_)/*ρ*_0_. The MR (Δ*ρ*/*ρ*) increases when the magnetic field increases gradually. At each temperature, the curves for the sample look very similar. But at *T =* 5 K, MR rises faster slightly than other higher temperature conditions. As shown in the black curve, the Δ*ρ*/*ρ* value is centered at 11.79% when the magnetic field is 4 T at a temperature of 300 K. When the temperature decreased at 5 K, keeping the same magnetic field of 4 T, the Δ*ρ*/*ρ* value increased to 38.35% (purple curves). These results experimentally suggest that the Δ*ρ*/*ρ* of Ag_2_Te NWs increased with the temperature decreasing gradually at the same magnetic field. Here, we also found a novel phenomenon that the magneto-resistance crosses over from a linear to a quadratic dependence on *H (T)* at the place of 4 T approximately. The Δ*ρ*/*ρ* shows a linear dependence on the low magnetic field (Figure 5b), but from the slope, we can notice that Δ*ρ*/*ρ* increases nonlinearly with increasing temperature at high *H*(*T*), which is different from the previous report [18,19]. We deduced that this novel phenomenon was caused by the nanostructure of the sample.

**Figure 6:**
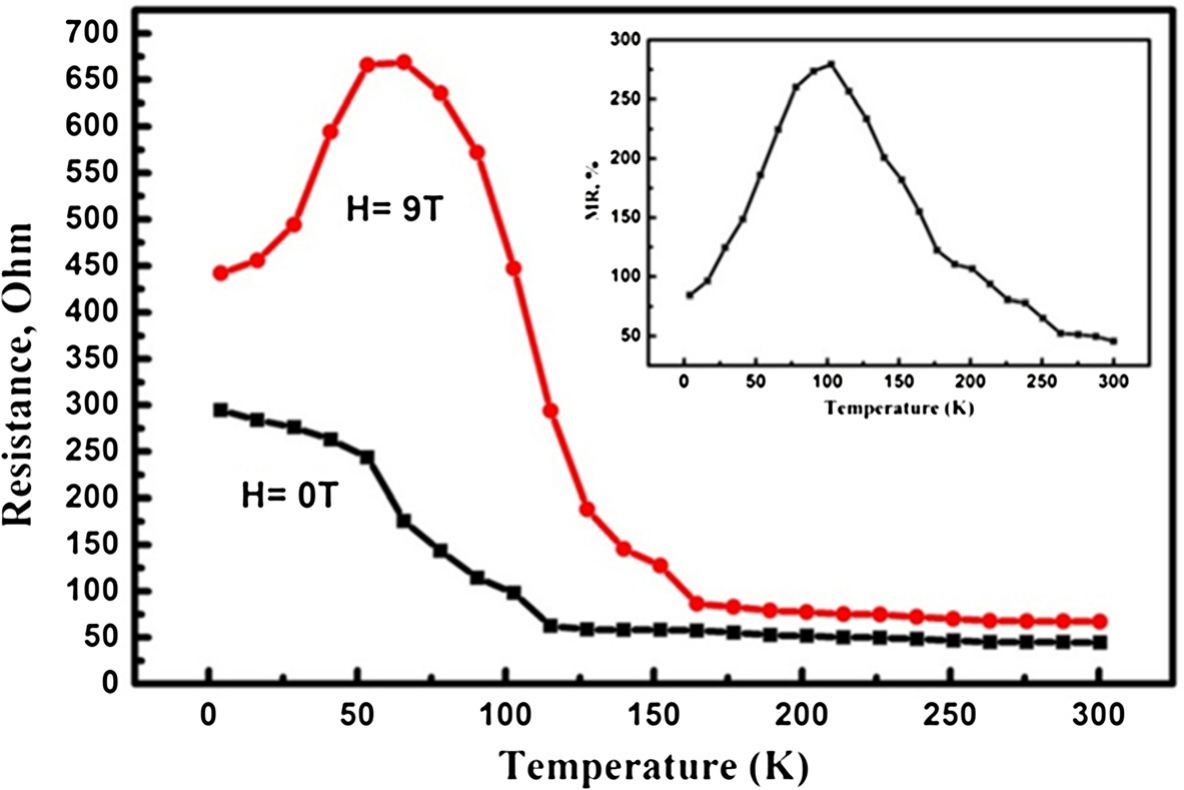
**Temperature dependence of resistivity of the as-prepared nanowires with and without magnetic field.** The inset shows the temperature dependence of MR of this sample.

Temperature-dependent MR of zero field (*R*_0_) and field (*R*_*H*_) resistivity is shown in Figure 6. The MR was calculated as MR = (*R*_*H*_ − *R*_0_) / *R*_0_, and the sample behavior was measured in temperature from 300 to 4 K. It is noteworthy that the resistivity measured by the magnetic field of 9 T becomes larger with the increasing magnetic field, and the field resistivity curve is peaked with a strong maximum at 66 K exhibited by the red line. Then, the product exhibits a steep decline of the resistivity with increasing temperature as illustrated in the figure. In contrast, no maximum peak was observed in the temperature-dependence curve of zero field resistivity of the Ag_2_Te nanowires, and the decrease of the resistance with decreasing temperature is pronounced (black line). At each temperature, the curves for the sample look very similar to the previous report [18]. However, comparing to the bulk [17] and thin film materials [18], we found that there is generally a larger change in *R*(*T*) as the sample size is reduced, which indicates that the size of the sample has a certain impact on the magneto-transport properties. While both field resistivity of 9 T and zero shows semiconductor characteristics at a high temperature region, it presents that resistivity is almost temperature-independent at a temperature more than 165 and 115 K, respectively. The inset shows the relative MR of as-synthesized nanowires. The MR amplitude increases from about 50% at room temperature to more than 250%. The MR also has a strong maximum at 100 K up to 280% corresponding to the maximum of the field resistance of 9 T. It was noted [18] that the classical picture seems incapable of explaining the silver chalcogenide data. That is why the search of analogies to other materials can be very helpful in understanding and explaining the observed phenomena. According to reports, the peak on the MR temperature curve of the Ag_2_Te nanowires suggests that grain boundary transport can play an important role in the MR effect in these materials [19]. Through analyzing the crystal structure of the monoclinic phase of Ag_2_Te [22], we know that this material can be considered a natural multilayered compound. Similar large positive MR was also discovered by Vernbank [29] et al. in nonmagnetic Cr/Ag/Cr trilayer structure. Nevertheless, more recently, a band calculation paper [14] by first principle calculations reported that β-Ag_2_Te is in fact a new binary topological insulator with gapless linear Dirac-type surface states. This raises the possibility that the observed unusual MR behavior can be understood from its topological nature and may largely come from the surface or interface contributions. This scenario is supported by the fact that experimental samples, doped with excess Ag, are granular materials [18,30], which makes the interface contribution significant. On the other hand, the highly anisotropic surface states may cause large fluctuation of mobility, which may also help to explain the unusual MR behavior [30]. To observe the unique electronic transport properties arising from the anisotropic Dirac cone, further experimental and theoretical studies are needed.

## Conclusions

In summary, a series of single crystalline 1D nanostructures of Ag_2_Te with well-controlled shapes and sizes were prepared by a facile one-pot hydrothermal synthesis approach. On the basis of these results, a rolling-up growth mechanism of the ultra-straight and long Ag_2_Te nanowires has been proposed. The formation of these 1D Ag_2_Te nanostructures can promote further studies and potential applications. Moreover, we systematically investigated the *I*-*V* characteristics and unusual MR behavior of the Ag_2_Te nanowires with monoclinic structure. It was found that the *I*-*V* of Ag_2_Te nanowires is more sensitive at low magnetic field, which reveals that the Ag_2_Te nanowires are suitable for low magnetic field sensor. In addition, the excellent single crystal quality with monoclinic structure raises the possibility for observing the unusual MR behavior in the as-prepared nanowires. Significantly, comparing to the bulk and thin film materials, we found that there is generally a larger change in *R*(*T*) as the sample size is reduced. This raises the possibility that the observed unusual MR behavior can be understood from its topological nature and may largely come from the surface or interface contributions.

## Abbreviations

1D: One-dimensional; AgNO3: Silver nitrate; Ag2Te: Silver telluride; EDS: X-ray energy dispersive analysis spectrum; FFT: Fast fourier transform; HRSEM: High-magnification SEM; HRTEM: High-resolution TEM; I-V: Current–voltage; MR: Magneto-resistance; N2H4 · H2O: Hydrazine hydrate; Na2TeO3: Sodium tellurite; NH3 · H2O: Ammonia; PPMS: Physical property measurement system; SAED: Selected area electron diffraction; SEM: Scanning electron microscopy; TEM: Transmission electron microscopy; TG-SDTA: Thermogravimetric and scalable differential thermal analysis; XPS: X-ray spectroscopy; XRD: X-ray diffraction.

## Competing interests

The authors declare that they have no competing interests.

## Authors’ contributions

GML designed and performed the fabrication and characterization experiments, analyzed the data, and drafted the manuscript. XBT performed the tests on the samples and helped in the drafting and revision of the manuscript. SMZ carried out current–voltage and magneto-resistance characteristics and critically revised the manuscript. NL conceived the study and helped in performing the experiment. XYY helped in the revision of the manuscript. All authors read and approved the final manuscript.

## Supplementary Material

Additional file 1: Figure A1 XRD spectra of the Ag_2_Te products under various growth times (3, 6, and 12 h reaction time) The XRD patterns reveal that these Ag_2_Te nanostructures have a monoclinic structure. Click here for file

Additional file 2: Figure A2 (a) XPS survey spectrum of the Ag_2_Te nanowires, and HRXPS in the (b) Ag 3d and (c) Te 3d regions. The molar ratio of silver to tellurium according to the quantification of peaks is 2.08:1.00, close to the stoichiometry of Ag_2_Te. Click here for file

Additional file 3: Figure A3. TG-DTA curves of the Ag_2_Te nanowires. From the DTA curve, it can be seen that the phase transition during the heating procedure occurred at 152°C, which confirms structural phase transition of Ag_2_Te. Click here for file

Additional file 4: Figure A4 Raman scattering spectrum of the as-prepared Ag_2_Te nanowires under different times of exposure. An interesting Raman scattering enhancement phenomenon has also been observed during the observation of Raman spectra. Click here for file
