# Supplementary material for: Morphological evolution, growth mechanism, and magneto-transport properties of silver telluride one-dimensional nanostructures
Source: Nanoscale Res Lett. 2013 Aug 20;8(1):356. doi: 10.1186/1556-276X-8-356 (PMC3765103; doi:10.1186/1556-276X-8-356)
Supplement: Additional file 1: Figure A1 — XRD spectra of the Ag2Te products under various growth times (3, 6, and 12 h reaction time) The XRD patterns reveal that these Ag2Te nanostructures have a monoclinic structure. [file 1556-276X-8-356-S1.doc]

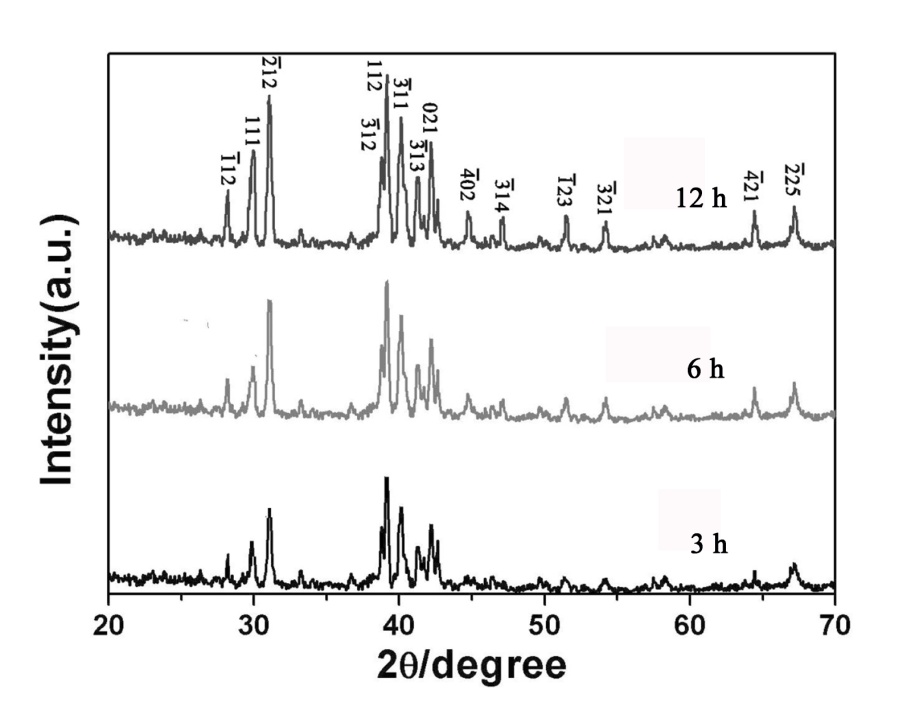


Figure A1. XRD spectra of the Ag_2_Te products under various growth time (3 h, 6 h and 12 h reaction time)

Generally, XRD patterns reveal that Ag_2_Te has a monoclinic structure. Concretely, all the visible peaks of samples match well with those of standard Ag_2_Te XRD pattern. No evidence of impurities can be found in the image. From the XRD patterns, we can know that the intensity of the XRD diffraction peaks is increasingly strong with the extension of reaction time. That is to say, crystallinity of Ag_2_Te samples is getting better and better in the heating process.
