# Supplementary material for: Morphological evolution, growth mechanism, and magneto-transport properties of silver telluride one-dimensional nanostructures
Source: Nanoscale Res Lett. 2013 Aug 20;8(1):356. doi: 10.1186/1556-276X-8-356 (PMC3765103; doi:10.1186/1556-276X-8-356)
Supplement: Additional file 2: Figure A2 — (a) XPS survey spectrum of the Ag2Te nanowires, and HRXPS in the (b) Ag 3d and (c) Te 3d regions. The molar ratio of silver to tellurium according to the quantification of peaks is 2.08:1.00, close to the stoichiometry of Ag2Te. [file 1556-276X-8-356-S2.doc]

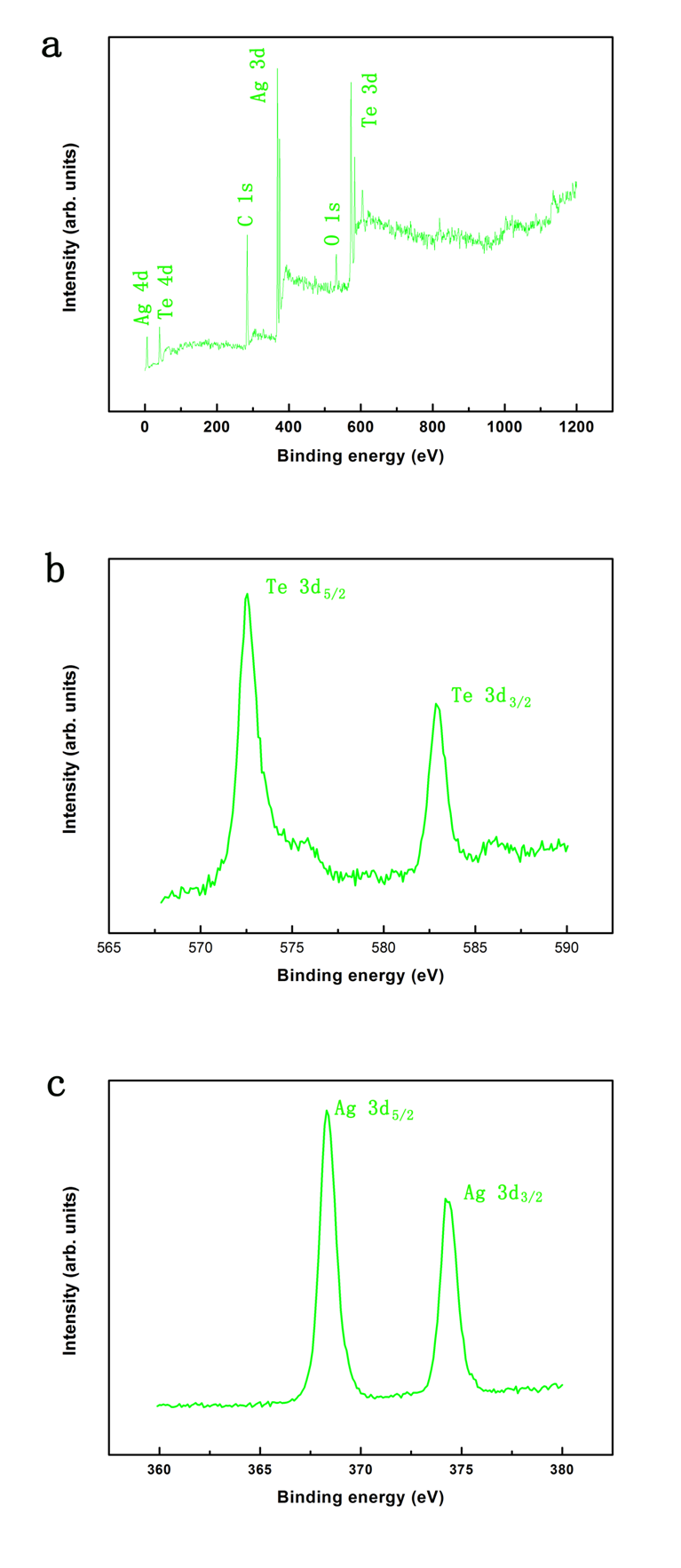


Figure A2. (a) XPS survey spectrum of Ag2Te nanowires, and HRXPS in the (b) Ag 3d and (c) Te 3d regions.

To understand the chemical composition of the sample, the XPS test was performed. As shown in Figure A2, the XPS spectra contain the wide-scan XPS spectrum (Figure A2a) with high-resolution XPS (HRXPS) spectra of Ag 3d (Figure A2b) and Te 3d (Figure A2c), respectively. From Fig. 3a, it's confirmed the as-synthesized products contain only Ag and Te elements because a little of the C and O comes from oil of the mechanical pump. Based on HRXPS, the peaks at 368.2 and 572.2 eV correspond to Ag 3d 5/2 and Te 3d 5/2, respectively, which indicate that the product is composed of Ag+ and Te2− valence states. Furthermore, as for Fig. 3c, two small peaks are observed at 576.1 and 586.5 eV, which can be attributed to Te (IV) oxide. Further, according to the quantification of HRXPS peaks, themolar ratio of Ag to Te is 1.986:1.00, close to the stoichiometry of Ag_2_Te.
