# Supplementary material for: Morphological evolution, growth mechanism, and magneto-transport properties of silver telluride one-dimensional nanostructures
Source: Nanoscale Res Lett. 2013 Aug 20;8(1):356. doi: 10.1186/1556-276X-8-356 (PMC3765103; doi:10.1186/1556-276X-8-356)
Supplement: Additional file 3: Figure A3. — TG-DTA curves of the Ag2Te nanowires. From the DTA curve, it can be seen that the phase transition during the heating procedure occurred at 152°C, which confirms structural phase transition of Ag2Te. [file 1556-276X-8-356-S3.doc]

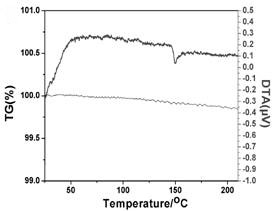


Figure A3. TG-DTA curves of Ag2Te nanowires.

To further ascertain the chemical compositions of the nanowires, the as-prepared products were examined by using TG-DTA, and the results are shown in Figure A3. As shown in Fig.A3, The TG curve shows that the product undergoes no weight-loss process from room temperature to 210 ° C, indicating that Ag2Te samples did not change in composition during the heating process. From the DTA curve, it can be seen that the phase transition during the heating procedure occurred at 152°C, which confirms structural phase transition of Ag2Te.
