# Supplementary material for: Morphological evolution, growth mechanism, and magneto-transport properties of silver telluride one-dimensional nanostructures
Source: Nanoscale Res Lett. 2013 Aug 20;8(1):356. doi: 10.1186/1556-276X-8-356 (PMC3765103; doi:10.1186/1556-276X-8-356)
Supplement: Additional file 4: Figure A4 — Raman scattering spectrum of the as-prepared Ag2Te nanowires under different times of exposure. An interesting Raman scattering enhancement phenomenon has also been observed during the observation of Raman spectra. [file 1556-276X-8-356-S4.doc]

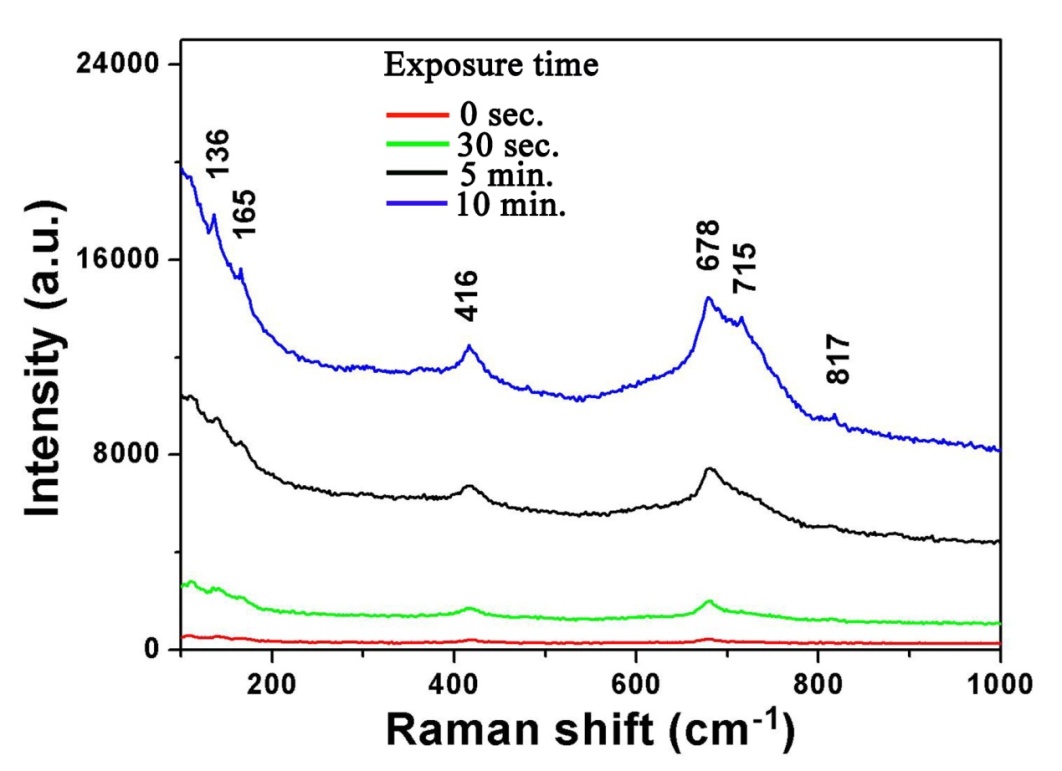


Figure A4. Raman scattering spectrum of the as-prepared Ag_2_Te products under different time of exposure.

Figure A4 shows the typical phonon Raman spectrum of the Ag_2_Te nanotubes under excitation of a 514.5 nm laser line. It is pronounced that the spectra of the Ag_2_Te nanotubes changed in the difference of the exposure time. The intensity of the Raman spectra of Ag_2_Te samples were rather weak at the beginning and the intensity of the Raman spectra became dramatically stronger, and the main peaks were sharpened when the exposure time was increased. Initially, the spectrum shows two weak peaks at 416 and 678 cm^-1^. As the exposure time is lengthened, four new peaks at 136, 165, 715 and 817 cm^-1^ appeared. An interesting Raman scattering enhancement phenomenon has also been observed during the observation of Raman spectra, in this regard, a tentative explanation of this phenomenon was proposed [A1,A2]. The Raman scattering was weak at the beginning because such laser-induced decomposition only produced very little TeO_2_ [A1,A2]. However, the concomitant appearance of silver still made it detectable by Raman. When the sample was exposed to the laser for a longer time, more and more silver atoms were generated, and the Raman intensity increased dramatically, which exhibited a significant enhancement of the Raman spectra [A1,A2].

**References**

A1. Qin A, Fang Y, Tao P, Zhang J, Su C: **Silver telluride nanotubes prepared by the hydrothermal method.** Inorg chem 2007, **46:**7403-7409.

A2. Samal A, Pradeep T: **Room-temperature chemical synthesis of silver telluride nanowires.** J Phys Chem C 2009, **113:** 13539-13544.
